# Comparative transcriptome analysis of wheat embryo and endosperm responses to ABA and H_2_O_2_ stresses during seed germination

**DOI:** 10.1186/s12864-016-2416-9

**Published:** 2016-02-04

**Authors:** Yonglong Yu, Shoumin Zhen, Shu Wang, Yaping Wang, Hui Cao, Yanzhen Zhang, Jiarui Li, Yueming Yan

**Affiliations:** College of Life Science, Capital Normal University, Beijing, 100048 China; Hubei Collaborative Innovation Center for Grain Industry, 434025 Jingzhou, China; College of Applied Sciences and Humanities of Beijing Union University, Beijing, 100083 China; Department of Plant Pathology, Kansas State University, Manhattan, KS 66506 USA

**Keywords:** Wheat, Embryo and endosperm, Seed germination, Transcriptome, ABA, H_2_O_2_

## Abstract

**Background:**

Wheat embryo and endosperm play important roles in seed germination, seedling survival, and subsequent vegetative growth. ABA can positively regulate dormancy induction and negatively regulates seed germination at low concentrations, while low H_2_O_2_ concentrations promote seed germination of cereal plants. In this report, we performed the first integrative transcriptome analysis of wheat embryo and endosperm responses to ABA and H_2_O_2_ stresses.

**Results:**

We used the GeneChip® Wheat Genome Array to conduct a comparative transcriptome microarray analysis of the embryo and endosperm of elite Chinese bread wheat cultivar Zhengmai 9023 in response to ABA and H_2_O_2_ treatments during seed germination. Transcriptome profiling showed that after H_2_O_2_ and ABA treatments, the 64 differentially expressed genes in the embryo were closely related to DNA synthesis, CHO metabolism, hormone metabolism, and protein degradation, while 121 in the endosperm were involved mainly in storage reserves, transport, biotic and abiotic stresses, hormone metabolism, cell wall metabolism, signaling, and development. Scatter plot analysis showed that ABA treatment increased the similarity of regulated patterns between the two tissues, whereas H_2_O_2_ treatment decreased the global expression similarity. MapMan analysis provided a global view of changes in several important metabolism pathways (e.g., energy reserves mobilization, cell wall metabolism, and photosynthesis), as well as related functional groups (e.g., cellular processes, hormones, and signaling and transport) in the embryo and endosperm following exposure of seeds to ABA and H_2_O_2_ treatments during germination. Quantitative RT-PCR analysis was used to validate the expression patterns of nine differentially expressed genes.

**Conclusions:**

Wheat seed germination involves regulation of a large number of genes involved in many functional groups. ABA/H_2_O_2_ can repress/promote seed germination by coordinately regulating related gene expression. Our results provide novel insights into the transcriptional regulation mechanisms of embryo and endosperm in response to ABA and H_2_O_2_ treatments during seed germination.

**Electronic supplementary material:**

The online version of this article (doi:10.1186/s12864-016-2416-9) contains supplementary material, which is available to authorized users.

## Background

Cereals are important to humankind, with over 2000 million tonnes harvested annually and used for food, livestock feed, and industrial raw materials. Wheat (*Triticum aestivum* L., 2n = 6x = 42, AABBDD), an allohexaploid species, is one of the most important and widely cultivated cereal crops and is a main food source for more than 40 % of the global population [1]. Wheat grains include mainly embryo and endosperm, and both play important roles in seed germination, seedling survival, and subsequent vegetative growth. The embryo forms radicle, plumule, and new plants, while the endosperm, which contains reserve substances, supplies nutrients for subsequent plant growth, which in turn affects wheat yield and quality.

Similar to most flowering plants, development and germination of wheat seeds are separated by a period of quiescence, which in many cases is also a dormancy phase. Only after breaking dormancy can the quiescent embryo germinate after imbibition. These processes have been investigated intensively at the physiological and molecular levels [2, 3]. Seed germination commences with imbibition, the uptake of water by the quiescent dry seed, and terminates with elongation of the embryonic axis [4]. Wheat seed germination undergoes a three-phase process of physiological and morphological changes, including a rapid initial uptake phase, a plateau phase, and a further water-uptake phase, corresponding to switches from the degradation of small-molecule sucrose to the metabolism of three major nutrients and photosynthesis [5]. These metabolic processes play key roles in seed germination by providing the required energy.

Abscisic acid (ABA) is a major hormone during seed germination [6]. The interactions among ABA, gibberellin (GA), ethylene, and brassinosteroids (BR) control the interconnected molecular processes of dormancy release and germination in eudicot seeds, such as *Arabidopsis* and tobacco [7, 8]. ABA is an important plant hormone that at low concentrations positively regulates dormancy induction and negatively regulates seed germination. ABA not only inhibits water uptake by preventing cell wall loosening of the embryo [9] but also specifically inhibits endosperm rupture rather than testa (i.e., seed coat) rupture [10]. Seeds undergo changes in both ABA content and sensitivity during germination in response to internal and external changes. Several studies to date have explored the roles of ABA during seed germination in both model plants and crop species such as *Arabidopsis* [11, 12], barley [13, 14], rice [15], lettuce [16], tomato [17], and coffee [18]. Particularly, most studies on the functions of ABA involved in seed germination have focused on the model plant *Arabidopsis*, including on the regulation of, and the protein kinases required for, ABA signaling during seed germination [19, 20] and transcriptional regulation of ABA-responsive genes in germinating seeds [21].

Treatment with hydrogen peroxide (H_2_O_2_) at low concentrations promotes seed germination of cereal plants, but a high H_2_O_2_ concentration limits germination of seeds, such as those of barley, wheat, and rice [22], *Arabidopsis* [11], pea [23, 24], maize [25, 26], *Zinnia elegans* [27], *Jatropha curcas* [28], and oat [29]. Endogenous H_2_O_2_ is generated in chloroplasts, mitochondria, and peroxisomes following exposure to a wide variety of abiotic and biotic stimuli [30]. Besides the important signaling function in response to environmental stimuli, H_2_O_2_ has toxic effects [31]. Catalase was proposed to be the most important H_2_O_2_-consuming enzyme in the presence of physiological concentrations of H_2_O_2_ [32]. The exogenous application of H_2_O_2_ can increase endogenous seed H_2_O_2_ content and cause carbonylation of storage proteins and several metabolic enzymes, thus enhancing seed germination [24]. Exogenously applied H_2_O_2_ ameliorates seed germination: one explanation is that the scavenging activity for H_2_O_2_ is high, resulting in the production of O_2_ for mitochondrial respiration [33, 34]. Another explanation is that H_2_O_2_ facilitates cracking of hard seeds, allowing them to interact with water [34].

In recent years, along with considerable progress in plant genomics, various transcriptomics and proteomics approaches have been used to investigate the mechanisms of seed germination and responses to various abiotic stresses in several plant species, such as *Arabidopsis* [35, 36], barley [37, 38], maize [39, 40], and rice [41, 42]. However, the majority of these studies focused on changes in the transcriptome of only one organ (embryo or endosperm) under one stress treatment, such as ABA or H_2_O_2_. An integrative transcriptome analysis of the responses of wheat embryo and endosperm to ABA and H_2_O_2_ stresses has not been reported to date.

In this study, we used the elite Chinese bread wheat cultivar Zhenmai 9023 and performed the first comparative transcriptome microarray analysis of the responses of embryo and endosperm to ABA and H_2_O_2_ treatments during seed germination using the GeneChip® Wheat Genome Array (Affymetrix, Santa Clara, CA). Numerous differentially expressed genes in embryo and endosperm responsive to ABA and H_2_O_2_ stresses involved in seed germination were identified. Our results provide novel insights into the molecular mechanisms of wheat seed germination and responses to abiotic stresses.

## Results

### Transcriptome expression profiling of the response to ABA and H_2_O_2_ stress during seed germination

The global transcriptome changes of embryo and endosperm in response to ABA and H_2_O_2_ treatments during wheat seed germination were investigated using the Affymetrix GeneChip® Wheat Genome Array. We grew wheat seeds in pure water as a contrast check (CK_embryo and CK_endosperm), while seeds in the experimental groups were separately treated with 100 mg/L ABA and 100 μmol H_2_O_2_ = (ABA_embryo and ABA_endosperm, H_2_O_2__embryo and H_2_O_2__endosperm). When seeds were imbibed in water for 16 h and the radicle emerged from episperm, we harvested the embryo and endosperm tissues separately in triplicate (18 individual samples). All microarray data from three biological replicates obtained in this study have been deposited in the NCBI GEO database. The normalized expression values obtained from three biological replicates based on independently treated plant materials are shown in Additional file 1.

**Fig. 1:**
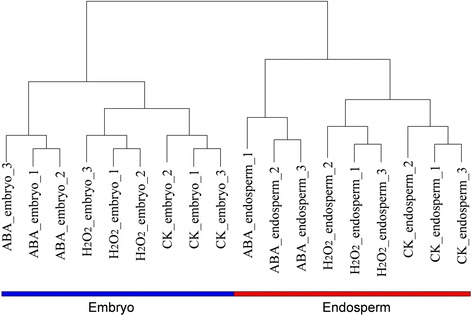
Hierarchical cluster dendrogram of normalized transcript abundances from 18 experiments including three biological replicates based on complete distance linkage. Two tissue fractions (E/A, red; Em, blue) were analyzed under different treatment (ABA, H_2_O_2_ and CK) during wheat germination

The degree of reproducibility was evaluated based on the square of the Pearson correlation coefficient, represented by the R^2^ value, between each biological replicate, of which all were larger than 0.99, indicating relatively good repeatability. All samples were substantiated by one unsupervised hierarchical clustering-based classification procedure, and the replicates were clustered as neighboring clades (Fig. 1).

The dendrogram showed major differences among all samples between embryo and endosperm during ABA and H_2_O_2_ treatment processes based on variations in transcribed gene sets (Fig. 1). The clustering of embryo and endosperm tissues suggests a smooth transition between different treatments in the same tissue. The two tissues showed distinct expression profiles, and tissue expression variability was greater than in biological treatments, showing clear tissue-specific expression. For example, the difference in gene expression patterns between ABA-embryo and ABA-endosperm was greater than that between ABA-embryo and CK-embryo, similar to the comparison of H_2_O_2_ and CK. Interestingly, excluding the relatively large expression differences between the two tissues, there was a marked difference between the ABA-treated group and the other two groups (H_2_O_2_-treated group and CK group; these were grouped under the second stratum of hierarchical clustering) in both embryo and endosperm samples. One possible explanation for the considerable changes is that H_2_O_2_ treatment more accurately mimics natural biological processes than ABA treatment.

To increase our understanding of the expression differences between embryo and endosperm under ABA and H_2_O_2_ treatments, we performed a heat map analysis of significant differentially expressed genes (Additional file 2). As shown in Additional file 3, the whole cluster was divided into three groups and marked with three colors (blue, yellow, and red) on the right side. In the blue group, endosperm genes were mostly downregulated, while those in embryo were upregulated. In the yellow group, most genes in both embryo and endosperm were upregulated under H_2_O_2_ treatment. However, in ABA-treated tissues, approximately 20 % of genes in embryo and 60 % in endosperm were upregulated. In the red group the genes in the ABA-treated tissues were downregulated, while those under H_2_O_2_ treatment were upregulated. In our results, we have found several genes which showing that the expression of the H_2_O_2_ samples is not significantly different but expression for these genes in the ABA samples is significantly different, such as BJ293360 (hormone metabolism), BJ296527 (Biodegradation of Xenobiotics), BJ299669 (storage proteins), CA719001 (starch cleavage), BQ169398 (starch cleavage) (Additional file 1 and Additional file 2). It verified the above results again on the other hand that H_2_O_2_ treatment was more accurately mimics natural biological processes than ABA treatment.

### Differential gene expression in embryo and endosperm in response to ABA and H_2_O_2_ treatments during seed germination

**Fig. 2:**
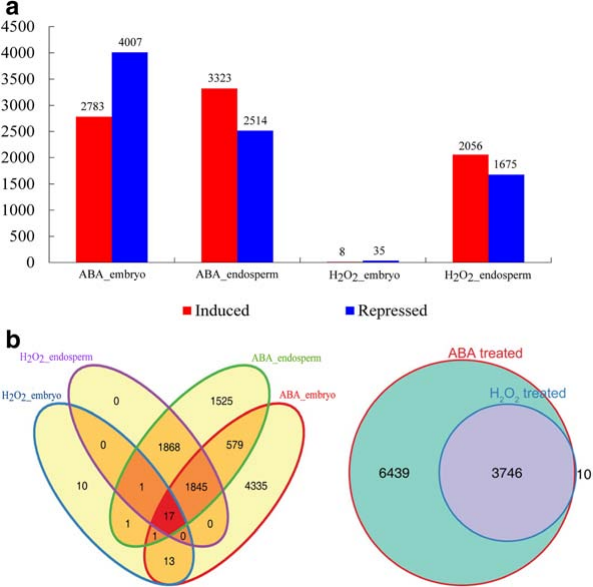
Differential gene expression in embryo and endosperm responsive to ABA and H_2_O_2_ treatments during seed germination. **a** Histogram of differentially expressed genes; **b** Venn diagram analysis of the differentially expressed genes under the ABA, H2O2 treatment compared to the control group. ABA treated means DEGs in the ABA_embryo and ABA_endosperm; H_2_O_2_ treated means H_2_O_2__embryo and H_2_O_2__endosperm. Figure 2B (*left*) is all the DEGs (*upregulated or downregulated*) that identified in four groups: ABA_embryo group, ABA_endosperm group, H_2_O_2__embryo group and H_2_O_2__ endosperm group shared with each other. Figure 2B (*right*) is 10,185 DEGs in the ABA-treated groups and 3756 in the H_2_O_2_-treated groups. 3746 DEGs were shared in ABA-treated groups and H_2_O_2_-treated groups

Significance analysis of microarrays (SAM) with a stringent 5 % false discovery rate (FDR) was applied to compare gene expression changes in embryo and endosperm under ABA and H_2_O_2_ treatments. The numbers of differentially expressed genes (DEGs) in embryo and endosperm in response to the treatments are shown in Fig. 2a and b. There are 6106 differentially expressed genes (DEGs) are regulated under the ABA treatment in the upregulated direction and 6521 DEGs in the downregulated direction compared to control groups. While in the H_2_O_2_ treated groups, there are 2064 DEGs in the upregulated direction and 1710 in the downregulated direction compared to control groups. In total, 4007 genes were downregulated while 2783 genes were upregulated in the ABA_embryo group, which showed that the sum total number (6790) of expression changes was similar to the ABA_endosperm (5837) containing 3323 upregulated and 2514 downregulated genes. Interestingly, the number of upregulated and downregulated genes under H_2_O_2_ treatment in embryo contrasted with those in endosperm (Fig. 2a). This sharp contrast suggested that the sensitivity of embryo and endosperm to H_2_O_2_ was different, and the embryo showed a different sensitivity in terms of response to ABA or H_2_O_2_ treatment. A greater number of genes were repressed than induced in embryo and there were more induced genes in endosperm exposed to ABA treatment (Fig. 2a). These results suggested that ABA repressed seed germination mainly by repressing embryo germination, while H_2_O_2_ induced seed germination mainly by activating endosperm genes.

Figure 2b (left panel) showed all the DEGs (upregulated or downregulated) that identified in four groups: ABA_embryo group, ABA_endosperm group, H_2_O_2__embryo group and H_2_O_2__ endosperm group shared with each other. We identified 10,185 DEGs in the ABA-treated groups and 3756 in the H_2_O_2_-treated groups (Fig. 2b right panel). There were 2442 DEGs not only in endosperm but also in embryo under ABA treatment, and 18 DEGs were identified in the above two tissues under H_2_O_2_ treatment. In total, 3746 DEGs were expressed in the above two treated tissues, of which 17 genes were differentially expressed in both embryo and endosperm under ABA and H_2_O_2_ treatments (Fig. 2b).

The key DEGs expressed under both ABA and H_2_O_2_ treatments in the embryo and endosperm are listed in Tables 1 and 2, respectively. In the embryo, 64 DEGs were associated mainly with DNA synthesis, CHO metabolism, hormone metabolism, and protein degradation. These functional groups belong to cellular processes and metabolic pathways. Half (50 %) of the 64 genes were assigned to cellular processes, 34 % to metabolic pathways, and only a few genes to development protein and hormone/signaling (Table 1). In the endosperm, 121 DEGs were closely related to the metabolism of storage reserves, transport, biotic and abiotic stresses, hormone metabolism, cell wall metabolism, signaling, and development. These functional classes are mostly involved in metabolic pathways, with about half of the assigned genes. The numbers of other assigned genes related to development proteins, transport, hormone/signaling, and cellular processes were similar (Table 2), all of which are important for seed germination and metabolic pathways. In the two gene lists, almost all of the genes were expressed only in embryo or endosperm, excluding CA602902 (related to stress defense) (Tables 1 and 2), which was expressed in both embryo and endosperm, suggesting that stress defense is important in both tissues during seed germination.

**Table 1:** Key differentially expressed genes in the embryo under ABA and H_2_O_2_ treatments

Genes	Functional classes		Fold-change	
	BINs	subBINs	ABA vs CK	H_2_O_2_ vs CK
CK212552	Metabolic pathways (assigned genes: 22)	PS.calvincyle. rubisco small subunit	1.6	3.53
AL820663		major CHO metabolism.degradation.starch.starch cleavage	0.083	0.18
CA720455		major CHO metabolism.degradation.starch.starch cleavage	0.21	0.35
BF293263		major CHO metabolism.degradation.starch.starch cleavage	0.3	0.41
CA644563		major CHO metabolism.degradation.starch.starch cleavage	0.14	0.23
CD452594		minor CHO metabolism.raffinose family.raffinosesynthases.putative	0.43	0.65
BQ166746		minor CHO metabolism.others	0.43	0.33
CD373448		minor CHO metabolism.others	2.39	1.54
CA741454		minor CHO metabolism.others	0.42	1.78
CD928919		cell wall.degradation.mannan-xylose-arabinose-fucose	0.34	0.57
BQ838005		cell wall.modification	0.46	0.37
AY491968		N-metabolism.ammonia metabolism.glutamine synthase	0.31	0.47
BJ263780		N-metabolism.ammonia metabolism.glutamine synthase	0.39	0.55
BJ266589		amino acid metabolism.synthesis.aromatic aa.tryptophan.indole-3-glycerol phosphate synthase	0.36	0.58
CK154440		secondary metabolism.phenylpropanoids	0.61	1.66
BJ231180		secondary metabolism.phenylpropanoids.lignin biosynthesis.PAL	0.29	1.53
BJ291883		misc.cytochrome P450	2.39	0
CA670423		misc.misc2	1.75	0.73
BQ169475		misc.gluco-, galacto- and mannosidases	0.46	0.52
BE429383		misc.gluco-, galacto- and mannosidases	0.44	0.64
CK215975		misc.oxidases - copper, flavone etc.	0.41	0.56
CA669389		misc.plastocyanin-like	6.05	1.68
CA602902	Development proteins (assigned genes: 3)	stress.biotic	0.2	0.54
CA602902		stress.biotic	0.22	0.53
BJ224721		development.unspecified	0.38	1.43
CA652678	Hormones and Signaling (assigned genes: 4)	hormone metabolism.ethylene.synthesis-degradation	0.28	0.58
CA484005		hormone metabolism.ethylene.signal transduction	2.6	1.88
CD928643		hormone metabolism.ethylene.signal transduction	0.48	0.65
BE604829		hormonemetabolism.jasmonate.synthesis-degradation.lipoxygenase	0.62	1.45
CD919701	Cellular processes (assigned genes: 32)	RNA.regulation of transcription.putative transcription regulator	19.23	0.12
BJ274465		DNA.synthesis/chromatin structure.histone	0.18	1.65
BJ225202		DNA.synthesis/chromatin structure.histone	0.13	1.67
BJ306445		DNA.synthesis/chromatin structure.histone	0.13	1.59
BJ217006		DNA.synthesis/chromatin structure.histone	0.14	1.65
BJ320258		DNA.synthesis/chromatin structure.histone	0.13	1.77
BJ308545		DNA.synthesis/chromatin structure.histone	0.21	1.69
CA719316		DNA.synthesis/chromatin structure.histone	0.16	1.58
BJ221397		DNA.synthesis/chromatin structure.histone	0.22	1.58
BJ231541		DNA.synthesis/chromatin structure.histone	0.31	1.56
BJ308545		DNA.synthesis/chromatin structure.histone	0.24	1.69
BJ320258		DNA.synthesis/chromatin structure.histone	0.17	1.53
BJ308450		DNA.synthesis/chromatin structure.histone	0.15	1.58
BJ219603		DNA.synthesis/chromatin structure.histone	0.2	1.61
CK209895		DNA.synthesis/chromatin structure.histone	0.19	1.57
BJ209178		DNA.synthesis/chromatin structure.histone	0.27	1.7
CK209895		DNA.synthesis/chromatin structure.histone	0.21	1.57
BJ214272		DNA.synthesis/chromatin structure.histone	0.27	1.57
BJ318011		DNA.synthesis/chromatin structure.histone	0.17	1.7
BJ229643		DNA.synthesis/chromatin structure.histone	0.28	1.61
CK217164		DNA.synthesis/chromatin structure.histone	0.21	2.02
BJ305771		DNA.synthesis/chromatin structure.histone	0.12	1.72
BJ277068		DNA.synthesis/chromatin structure.histone	0.38	1.63
BJ306762		DNA.synthesis/chromatin structure.histone	0.46	1.49
BJ308450		DNA.synthesis/chromatin structure.histone	0.15	1.61
BJ306762		DNA.synthesis/chromatin structure.histone	0.44	1.44
BJ207229		DNA.synthesis/chromatin structure.histone	0.5	1.37
CA712796		protein.postranslational modification	0.39	0.62
BQ169109		protein.degradation.cysteine protease	0.47	0.21
AJ612538		protein.degradation.cysteine protease	0.4	0.27
CK208190		protein.degradation.serine protease	0.5	0.41
BJ296643		protein.degradation.ubiquitin.E2	2.31	0.55
BF200045	Miscellanious (assigned genes: 3)	metal handling. binding.chelation and storage	0.48	0.55
CA729143		metal handling.binding. chelation and storage	0.36	0.36
CA666515		metal handling.binding. chelation and storage	0.34	0.38

**Table 2:** Key differentially expressed genes in the endosperm under ABA and H_2_O_2_ treatments

Genes	Functional classes		Fold-change	
	BINs	subBINs	ABA vs CK	H_2_O_2_ vs CK
CK161429	Metabolic pathways (assigned genes: 57)	PS. lightreaction. ATP synthase	2.59	2.14
BJ247599		major CHO metabolism. degradation. sucrose.invertases. cell wall	0.31	0.41
CD869165		major CHO metabolism. degradation. sucrose.Susy	0.44	0.4
CA639484		glycolysis. TPI	0.19	0.3
CA605311		fermentation. ADH	0.36	0.33
CD868238		fermentation. ADH	0.27	0.3
BQ166030		fermentation. ADH	0.29	0.32
CA681784		fermentation. aldehyde dehydrogenase	2.37	2.16
CD891243		fermentation. aldehyde dehydrogenase	2.67	4.36
CA720946		TCA / org. transformation. TCA.CS	2.21	2.44
CK198230		cell wall. precursor synthesis. phosphomannomutase	0.5	0.49
BJ234908		cell wall. precursor synthesis. MUR4	2.02	2.23
CD452786		cell wall. cell wall proteins. AGPs	0.26	0.49
CA611920		cell wall. modification	0.42	0.36
CA638337		cell wall. modification	0.45	0.45
CA644687		cell wall. modification	0.43	0.47
BJ222865		lipid metabolism. Phospholipid synthesis. choline-phosphate cytidylyltransferase	2.23	2.2
CA720390		lipid metabolism. Phospholipid synthesis. cyclopropane-fatty-acyl-phospholipid synthase	3.08	0.51
CA610745		lipid metabolism. “exotics” (steroids, squaleneetc). sphingolipids	1.88	2.57
BJ297605		N-metabolism. ammonia metabolism. glutamine synthase	2.04	1.57
BE443630		amino acid metabolism. synthesis.central amino acid metabolism. GABA. GABA transaminase	4.8	2.61
BQ280449		amino acid metabolism. Synthesis.central amino acid metabolism. GABA. GABA transaminase	3.75	2.02
BE585584		amino acid metabolism. synthesis.central amino acid metabolism. GABA. GABA transaminase	4.09	2.28
CD892913		amino acid metabolism.synthesis.central amino acid metabolism.GABA.Glutamate decarboxylase	0.2	0.48
BT009245		amino acid metabolism.synthesis.aspartate family.asparagine	3.61	2.26
BJ243273		amino acid metabolism.degradation.aromatic aa.tyrosine	2.56	2.7
CD935642		amino acid metabolism.degradation.aspartate family.methionine.methionine gamma-lyase	4.19	2.82
CD492002		secondary metabolism.wax	0.23	0.34
CA599972		secondary metabolism.isoprenoids.mevalonate pathway.HMG-CoA synthase	2.08	2.37
CA599972		secondary metabolism.isoprenoids.mevalonate pathway.HMG-CoA synthase	2.85	2.87
BJ285801		secondary metabolism.phenylpropanoids.lignin biosynthesis.PAL	1.94	2.1
CA640772		secondary metabolism.phenylpropanoids.lignin biosynthesis.CAD	2.21	2.04
CK193717		secondary metabolism.phenylpropanoids.lignin biosynthesis.HCT	2.55	2.14
CA676957		secondary metabolism.N misc.alkaloid-like	1.82	2.51
BJ288127		secondary metabolism.flavonoids.dihydroflavonols.dihydrokaempferol 4-reductase	2.58	2.07
CA653342		C1-metabolism.dihydroneopterinaldolase	0.44	0.53
CA641602		tetrapyrrole synthesis.ferrochelatase	2.63	3.77
BJ244873		nucleotide metabolism.phosphotransfer and pyrophosphatases.misc	2.05	4.31
BJ320066		misc.UDP glucosyl and glucoronyltransferases	2.07	2.65
BJ250503		misc.UDP glucosyl and glucoronyltransferases	1.98	2.05
BJ292155		misc.gluco-, galacto- and mannosidases	2.35	2.61
BJ292155		misc.gluco-, galacto- and mannosidases	2.49	2.73
BJ208962		misc.oxidases - copper, flavone etc.	0.34	0.45
CA733231		misc.glutathione S transferases	2.4	2.24
AY064480		misc.glutathione S transferases	0.37	0.44
CA733223		misc.cytochrome P450	2.15	3.13
CK169303		misc.cytochrome P450	2.19	2.61
AF031195		misc.plastocyanin-like	2.36	2.15
BJ283868		misc.protease inhibitor/seed storage/lipid transfer protein (LTP) family protein	0.35	0.49
BQ162656		misc.protease inhibitor/seed storage/lipid transfer protein (LTP) family protein	0.35	0.43
BQ838076		misc.protease inhibitor/seed storage/lipid transfer protein (LTP) family protein	0.15	0.26
BQ170864		misc.protease inhibitor/seed storage/lipid transfer protein (LTP) family protein	3.01	0.48
BQ838076		misc.protease inhibitor/seed storage/lipid transfer protein (LTP) family protein	0.13	0.24
CD453971		misc.acid and other phosphatases	1.97	2
CA662320		misc.short chain dehydrogenase/reductase (SDR)	2.65	2.43
BJ280289		misc.short chain dehydrogenase/reductase (SDR)	2.04	2.44
CA719923		misc.GDSL-motif lipase	0.46	2.98
CA608846	Development proteins (assigned genes: 18)	stress.biotic	1.94	2.43
AF112966		stress.biotic	2.36	1.88
CA721939		stress.biotic	3.56	2.99
CA602902		stress.biotic	0.11	0.15
CD453602		stress.biotic	0.2	0.43
CA602902		stress.biotic	0.097	0.15
CA611464		stress.abiotic.heat	2.11	2.18
CD373694		stress.abiotic.drought/salt	0.3	0.26
CA696591		stress.biotic.PR-proteins	2.21	2.75
CK216297		stress.abiotic.unspecified	0.42	0.25
CK216158		stress.abiotic.unspecified	1.63	2.45
CK199583		development.late embryogenesis abundant	0.47	0.26
CA604393		development.unspecified	1.8	2.35
CA639698		development.unspecified	1.84	2.54
BJ267646		development.unspecified	4.1	2.05
BJ284862		development.unspecified	0.48	0.4
BQ161132		development.unspecified	2.54	3.05
BJ274121		development.unspecified	0.41	0.38
CA728921	Transporting (assigned genes: 11)	transporter.sugars	2.84	1.92
BJ286275		transport.amino acids	0.38	0.39
CA663166		transport.potassium	2.13	2.54
CN012655		transport.metabolite transporters at the mitochondrial membrane	2.54	2.64
CA647258		transport.metabolite transporters at the mitochondrial membrane	1.93	2.02
CA647258		transport.metabolite transporters at the mitochondrial membrane	2.63	2.49
CN012655		transport.metabolite transporters at the mitochondrial membrane	2.38	3.1
CA641308		transport.ABC transporters and multidrug resistance systems	2.96	2.17
CA640534		transport.ABC transporters and multidrug resistance systems	2.77	2.62
CA719491		transport.misc	2.07	3.27
BJ267436		transport.misc	2.21	2.34
CA695230	Hormones and Signaling (assigned genes: 13)	hormone metabolism.gibberelin.synthesis-degradation	3.32	3.47
BT008949		hormone metabolism.auxin.signal transduction	0.38	0.47
CD452527		hormone metabolism.ethylene.induced-regulated-responsive-activated	0.28	0.45
CD866886		hormone metabolism.ethylene.induced-regulated-responsive-activated	0.44	0.26
CA669976		hormone metabolism.jasmonate.synthesis-degradation.12-Oxo-PDA-reductase	2.09	2.2
CD373926		hormone metabolism.jasmonate.synthesis-degradation.12-Oxo-PDA-reductase	1.87	3.19
CD492184		signalling.receptor kinases.leucine rich repeat III	0.56	0.53
CA614315		signalling.calcium	0.09	0.23
CA614315		signalling.calcium	0.075	0.24
BJ268280		signalling.calcium	2.38	3.8
CA608459		redox.thioredoxin	1.66	2.25
CA607898		redox.ascorbate and glutathione.glutathione	2.3	2.93
BE426829		redox.ascorbate and glutathione.glutathione	0.36	1.99
CA619551	Cellular processes (assigned genes: 20)	RNA.transcription	0.35	0.4
CA741924		RNA.regulation of transcription.bHLH,Basic Helix-Loop-Helix family	0.2	0.45
CD453519		RNA.regulation of transcription.WRKY domain transcription factor family	1.64	1.98
CA611439		RNA.RNA binding	1.78	1.79
D37943		DNA.synthesis/chromatin structure.histone	0.48	0.39
D38090		DNA.synthesis/chromatin structure.histone	0.38	0.35
D38087		DNA.synthesis/chromatin structure.histone	0.37	0.41
CA674072		protein.synthesis.ribosomal protein.prokaryotic.chloroplast.30S subunit.S11	0.62	0.57
CA697403		protein.synthesis.ribosomal protein.prokaryotic.chloroplast.30S subunit.S19	0.43	0.55
CA716133		protein.postranslational modification	1.93	2.2
CA655039		protein.postranslational modification	3.09	2.81
BJ227360		protein.postranslational modification	0.41	0.47
BJ240977		protein.postranslational modification	2.7	2.27
BJ274189		protein.postranslational modification	0.25	0.44
BQ161936		protein.postranslational modification	0.39	0.44
CA606579		protein.degradation	1.88	2.14
BJ251154		protein.degradation	0.32	2.71
CA699722		protein.degradation	1.68	4.28
CA642033		protein.degradation.ubiquitin.E3.SCF.cullin	2.14	2.34
BQ246681		cell. organisation	2.68	2.74
BQ806459	Miscellanious (assigned genes: 2)	Biodegradation of Xenobiotics.lactoylglutathionelyase	5.29	2.3
BQ806459		Biodegradation of Xenobiotics.lactoylglutathionelyase	5.61	2.3

**Fig. 3:**
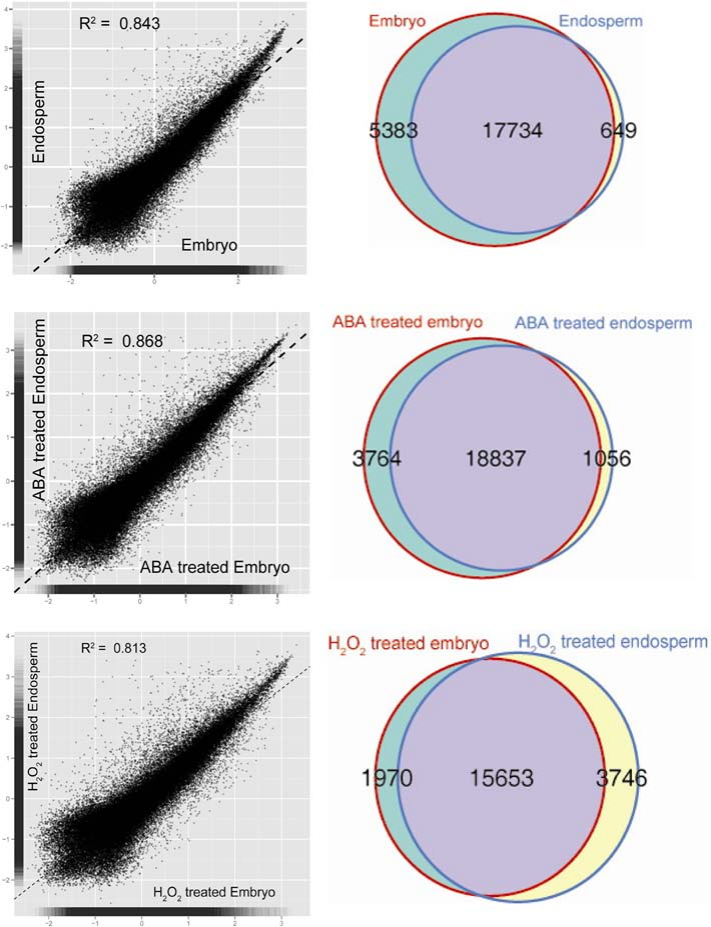
Comparison of the embryo and endosperm transcriptome of the control and treatment group. (*Left list*) Plot of Z-score transformed embryo versus endosperm expression data shows similar expression of most genes in both tissues. (*Right list*) The number of detected expressed genes in embryo, endosperm, or both tissues

Embryo and endosperm transcript levels, determined based on Z-score transformation, are shown in scatter plots (Fig. 3). This transformation was used to compare the expression levels between two tissues. With ABA treatment, a greater number of genes were expressed in both embryo and endosperm compared to under CK and H_2_O_2_ treatments. ABA treatment increased the similarity of the gene expression patterns between the two tissues: R^2^ = 0.868 in the ABA treatment compared to 0.843 in the CK. The H_2_O_2_-regulated group showed decreased global expression similarity compared to ABA treatment.

### MapMan analysis

To further investigate the transcriptome changes of embryo and endosperm in response to ABA and H_2_O_2_ treatments during seed germination, we compared the transcriptome data using MapMan software, which is a user-driven tool for mapping transcriptome data, define functional categories, and identify significantly overrepresented functional groups.

**Fig. 4:**
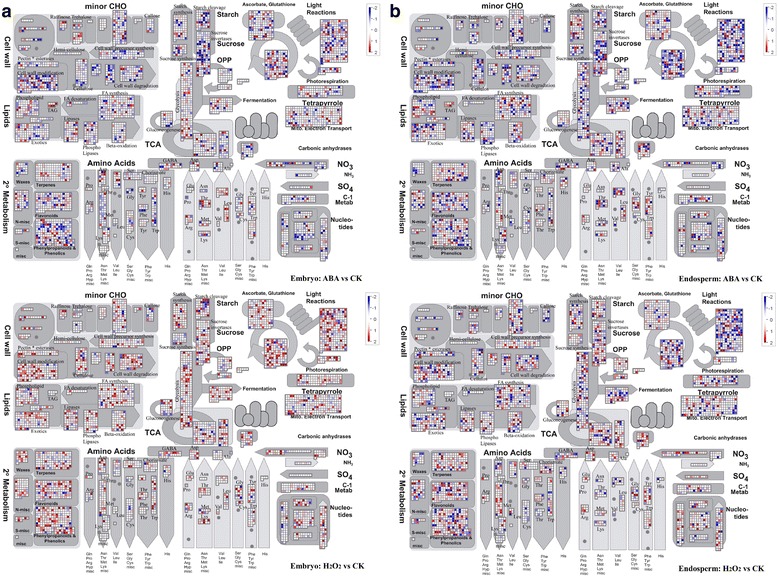
MapMan metabolism overview maps showing differences in transcript levels between ABA/H_2_O_2_ treatment and CK during seed germination. **a** ABA vs CK and H_2_O_2_ vs CK in the embryo. **b** ABA vs CK and H_2_O_2_ vs CK in the endosperm. Log2 ratios for average transcript abundance were based on three replicates of AffymetrixGeneChip ^*®*^ Wheat Genome Array. The resulting file was loaded into the MapMan Image Annotator module to generate the metabolism overview map. On the logarithmic color scale, blue represents downregulated transcripts, and red represents upregulated transcripts

During seed maturation, reserve materials such as starch, sucrose, lipids, and storage proteins are gradually accumulated. Metabolic activities within the seed are significantly downregulated during dormancy and then reactivated during germination. Many hormones—including ABA, GA, H_2_O_2_, BR, ethylene, auxin (IAA), and jasmonate (JA)—are involved in seed germination. As shown in Fig. 4, the gene expression profiles during seed germination under ABA and H_2_O_2_ treatments, respectively, were determined. H_2_O_2_ treatment resulted in the upregulation of genes involved in seed germination in both embryo and endosperm. In contrast, a greater number of genes were expressed under ABA treatment and showed downregulated expression pattern. This indicated that H_2_O_2_ could promote seed germination, while ABA represses seed germination. According to our results, H_2_O_2_ treatment affected the expression of only a small number of genes in the embryo (Fig. 2a), but these genes caused marked effects and changes (Fig. 4). These effects and changes were mainly in glycolysis, sucrose degradation, cell wall, lipid metabolism, and photosynthesis (Fig. 4a and b). Lipids are found in the embryo, and genes associated with FA synthesis and beta-oxidation were largely upregulated in H_2_O_2_-treated embryos. Indeed, genes involved in photosynthesis, such as light reactions and photorespiration, were strongly upregulated. Another interesting result is that genes related to ascorbate and glutathione were upregulated in H_2_O_2_-treated embryo compared to the endosperm. This suggested that embryo is more sensitive to oxidative stress than endosperm.

The accumulated reserve materials of wheat seeds are stored mainly in the seed endosperm. During seed germination, they begin to metabolize along with imbibition. Sucrose, one type of deposit, is a micromolecule substance that is easily mobilized. Our results showed that the inhibition of ABA and the ability of H_2_O_2_ to promote sucrose mobilization were most distinct in embryo and endosperm (Fig. 4). The majority of genes related to sucrose mobilization were downregulated under ABA treatment, while several were upregulated under H_2_O_2_ treatment. In the process of sucrose degradation, sucrose synthase (SUSY) is the most important enzyme, and the related key gene to SUSY is CA623473 (Additional file 1). Other enzymes, such as hexokinase and fructokinase, also play a role. Besides energy provision, sucrose acts as transmembrane transporter in the cell membrane.

The main reserve deposit of wheat seeds is in the form of starch, which is mainly present in endosperm. Different treatments result in distinct changes in starch cleavage and synthesis in endosperm. Two important enzymes, amylase for wheat starch cleavage and UDP-glucose pyrophosphorylase (UGPase) for starch synthesis, are required during metabolic processes. The majority of genes related to starch cleavage were downregulated, while those for starch synthesis were upregulated under ABA treatment. However, H_2_O_2_ treatment resulted in the opposite changes (Fig. 4). Glycolysis and the TCA cycle are very important metabolic pathways during seed germination. Based on our results, the expression levels of genes related to both glycolysis and the TCA cycle in endosperm were higher than those in embryo, indicating that the primary pathways occurred in endosperm to provide energy for wheat seed germination.

During seed germination, rapid water absorption leads to seed expansion and penetration of the embryonic axis. According to our gene expression data, genes related to major constituents of the cell wall (cellulose, pectin, hemi-cellulose, and expansins) were activated during imbibition. This suggests that the cell wall was undergoing continuous modification and synthesis; however, it was also experiencing continuous degradation. Several important enzymes related to degradation such as cellulases and pectatelyases are activated early during seed germination. Hence, genes associated with cell wall synthesis and degradation were activated during the process of imbibition. We speculated that the majority of the fractured cell wall might be degraded into small molecules to provide raw materials for cell wall synthesis during seed germination. However, our gene expression data showed that most cell wall-related genes were upregulated under H_2_O_2_ treatment and downregulated under ABA treatment. Furthermore, these related genes were more activated in H_2_O_2_-treated embryos than other treatments (Fig. 4).

As shown in Fig. 4a, genes related to photosynthetic processes, such as the light reactions and the Calvin cycle, became activated in the embryo. However, in the endosperm, the photosynthesis-related genes remained inactivated (Fig. 4b). This suggested that photosynthesis genes were activated first in the embryo, and that their expression was promoted by H_2_O_2_.

### Verification of gene expression patterns using qRT-PCR

**Fig. 5:**
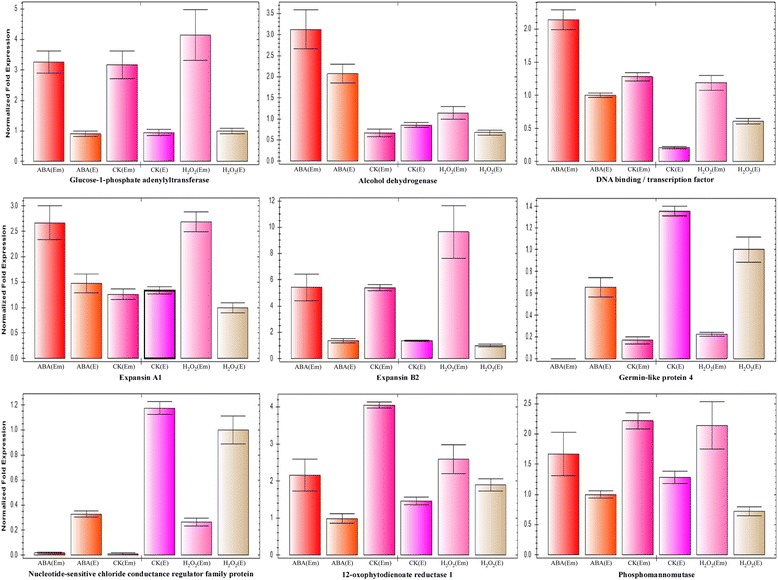
Verification of 9 key gene expression patterns by qRT-PCR. The horizontal axis is the different treatment in the different tissues (6 groups) during seed germination, and the vertical axis is the expression of each group after normalized fold

Quantitative real-time polymerase chain reaction (qRT-PCR) with specific primers was used to confirm the expression of nine representative genes (Additional file 4). These genes are involved in starch synthesis, fermentation, RNA regulation of transcription, cell wall modification and precursor synthesis, abiotic stress, transport, and hormone metabolism, and play pivotal roles in seed germination. Optimization experiments showed higher amplification efficiency and specificity of nine targeted genes (Additional file 5). As shown in Fig. 5, the expression patterns of five genes (CD491559, CA498269, AY543540.1, Y09916.1, and CK198230) were consistent with those determined by transcriptome microarray analysis. The expression patterns of the other four genes (BJ249131, AY485121.1, BQ170546, and CA645154) generally followed the transcriptional expression models.

## Discussion

### Tissue differential expression and significant functional classes in wheat embryo and endosperm in response to ABA and H_2_O_2_ treatments

Our results showed that embryo and endosperm exhibit different responses when exposed to ABA and H_2_O_2_ treatments. Treatment with a low concentration of H_2_O_2_ facilitates seed germination, while low concentrations of ABA repress seed germination. Thus, there is tissue differential expression during seed germination in response to ABA and H_2_O_2_ treatments, similar to in germinating *Arabidopsis* seeds [43]. Heat map analysis of differential genes showed that genes are expressed differentially not only in number, but also in classification, between embryo and endosperm (Additional file 3). During seed germination, the key functional class in embryo is cellular processes, while that in endosperm is metabolic pathways. These results suggest that genes in embryo and endosperm show tissue-differential expression.

According to our results, lipid degradation is repressed by ABA in the embryo, but not obvious in endosperm tissues. In endosperm, ABA inhibited mainly storage reserve metabolism (Fig. 4b). Apparently, a tissue-specific response to ABA sensitivity exists between embryo and endosperm. This differential sensitivity of lipid mobilization to ABA in the embryo and endosperm was confirmed in *Arabidopsis* and tobacco seeds [12, 44], suggestive of wide conservation and functional differentiation of embryo and endosperm among seed plants.

In this study, we detected several significant functional classes in embryo and endosperm during seed germination based on analysis of differentially expressed genes. We hypothesize that the cellular processes are more important than other functional classes in embryo during seed germination, as suggested by the assigned gene numbers, similar to the metabolic pathways in endosperm. Transport, a functional class important to metabolism, was present only in endosperm based on the classification of differentially expressed genes (Table 2). This suggested that endosperm, the main tissue for energy supply, could provide the energy for seed germination through various metabolic pathways. Small molecules generated during the germination process were transported to the embryo and other locations to support germination processes, such as DNA synthesis, bud germination, and development. Another difference between embryo and endosperm is that the cell wall-related genes in embryo were involved in degradation and modification, while those in endosperm were involved in synthesis and modification. This may be because penetration of the embryonic axis occurs in embryo during seed germination, and nutrients are required. According to our results, only CA602902 was expressed in embryo and endosperm (Tables 1 and 2). The CA602902 gene was involved in biotic stress responses, and similar genes (prp4, At3g19690, Os07g0129200) have been found in maize [45], *Arabidopsis* [46], and rice [47].

The endosperm in mature cereal seeds of comparatively large sizes is important to understanding the regulation of seed germination. In wheat, endosperm accounts for about 90 % of the whole seed and plays a vital role in seed germination. According to this study, an important role of endosperm in wheat seeds during imbibition is to provide energy for seed germination and the post-germination period. In the seeds, hydrolytic enzymes are secreted from the aleurone layer into the free endosperm to mobilize starch, protein, and lipid reserves. Carbon in the form of sucrose from endospermic reserves is transported to the embryo to fuel post-germinative growth [48]. Similar results have been reported in other angiosperm seeds, such as *Arabidopsis* [43] and barley [49]. Our results demonstrated that ABA repressed seed germination by inhibiting the activity of hydrolytic enzymes such as amylase, hexokinase, PPFK, and PK during reserve mobilization. A previous study also showed that an ABA-induced protein kinase could mediate ABA suppression of amylase expression [50].

Our results demonstrated that activation of cell wall genes was associated with seed expansion and penetration of the embryonic axis during seed imbibition. ABA repressed the expression of genes related to the cell wall, while H_2_O_2_ induced the expression of these genes in both embryo and endosperm. Endosperm is considered a barrier to radicle protrusion in many angiosperm seeds. Furthermore, endosperm weakening can mediate control of radicle protrusion during *Brassicaceae* seed germination [10]. The other functions of endosperm were to control germination by secreting cell wall-loosening enzymes such as β-1, 4-glucanase, polygalacturonase, and expansins to degrade cell walls of the endosperm and seed coat, thus removing mechanical barriers to radicle emergence [12, 43]. According to our transcriptome data, β-1,4-glucanase and polygalacturonase are two important cell wall-degradative enzymes, named cellulase and pectinase, respectively (Additional file 1). Expansins facilitate cell wall extensions, possibly by disrupting hydrogen bonding between hemicellulosic wall components and cellulose microfibrils [51]. Activation of the genes related to these three types of enzymes results in cell wall degradation, modification, and synthesis. Thus, we hypothesize that ABA/H_2_O_2_ repressed/induced seed germination by inhibiting/facilitating gene expression of these enzymes.

In tobacco, the micropylar endosperm region could function as a water reservoir for the embryo [44]. Wheat, similar to tobacco, contains comparatively large endosperms in mature seeds. It is likely that wheat endosperm plays a unique role in preserving water.

### Signaling function and regulation of H_2_O_2_

Our results showed that exogenous application of H_2_O_2_ could promote seed germination, as has been reported previously [11]. H_2_O_2_ has two important roles: serving as a signal in response to environmental stimuli and regulating hormonal metabolism, with effects on accelerating seed germination [11, 52].

Our recent work showed that when placed in water, wheat seeds activate a series of mechanisms that respond to biotic and abiotic stresses during germination due to changes in the external environment [5]. Mitogen-activated protein kinase (MAPK) is believed to play a key role in these biotic and abiotic responses. MAPKs receive hormonal and other signals, and mediate transcription factors through the MAPK cascade reaction. These transcription factors then regulate defensive genes encoding stress-related proteins that function in the responses to external biotic and abiotic stresses [5]. The regulation of defensive genes can protect germinating seeds against damage from biotic and abiotic stresses. Similar results have been reported in *Arabidopsis* [53], rice [54], and tobacco [55]. H_2_O_2_ serves as a second messenger in cellular signal transduction pathways, and can lead to the activation of MAPKs [24, 56, 57]. Therefore, we propose that H_2_O_2_ promotes seed germination by regulating the activation of MAPKs. AtMPK6, an *Arabidopsis* MAPK, is involved in signal transduction pathways responding to these biotic and abiotic stresses for reactive oxygen species (ROS) [58, 59]. In the present study, we identified a gene (AY173962.1) that is similar to At2g43790, which encodes AtMPK6; the encoded protein may have the same function as AtMPK6.

Two major plant hormones, ABA and GA, play an important role in controlling wheat seed germination. Both ABA and GA are under the regulation of H_2_O_2_ in seed dormancy and germination [11]. H_2_O_2_ upregulates ABA catabolism, resulting in a decreased ABA content, and promotes GA biosynthesis during imbibition, while ABA plays an important role in enhancing seed dormancy and delaying germination. Hence, the decreased ABA content could benefit seed germination, which indirectly shows that H_2_O_2_ promotes seed germination. Exogenous H_2_O_2_ can increase ABA catabolism by enhancing the expression of CYP707A genes in *Arabidopsis* [11]. CYP707A1 encoded by At4g19230, a member of the CYP707A gene family, may play an important role in determining ABA levels. In our study, we identified an important gene, BJ291883 (Table 1), which may have the same function. GA has an antagonistic role with ABA in seed germination. For example, our results showed that ABA suppressed the expression of amylase, while GA induces transcription of amylase in cereal seeds [50, 60, 61]. GA was found to promote seed germination in many species, such as *Arabidopsis* [62, 63] and maize [64].

### Reactive oxygen species (ROS) in seed germination

As byproducts of aerobic metabolism, reactive oxygen species (ROS) such as H_2_O_2_, O_2_^−^, hydroxyl radicals, and superoxide radicals are produced during seed germination. The accumulation of ROS not only leads to cell injury and disturbances in seed germination but also functions as a signaling molecule and is involved in a wide range of responses to various stimuli [65]. The balance between ROS production and scavenging regulates their accumulation, and antioxidative mechanisms are important for the scavenging of ROS. Levels of antioxidant compounds, such as ascorbate and glutathione, increase during wheat and *Pinuspinea* seed germination [66, 67]. Similar results were found in our study: two differentially expressed genes (CA607898 and BE426829) related to ascorbate and glutathione were detected in endosperm (Table 2). The expression levels of CA607898 and BE426829 increased by about two to three-fold under H_2_O_2_ treatment, indicating that detoxifying enzymes and antioxidant compounds were strongly expressed, possibly due to increased H_2_O_2_ toxicity. ABA-treated endosperm showed upregulation of CA607898 and downregulation of BE426829, possibly because of the different results of ABA signal transduction affected by H_2_O_2_.

## Conclusions

In this study, we performed a global transcriptome profiling analysis using the Affymetrix GeneChip® Wheat Genome Array to characterize gene expression changes in embryo and endosperm in response to ABA and H_2_O_2_ treatments during wheat seed germination. Microarray analysis enabled detection of a large number of genes in germinating seeds related to ABA and H_2_O_2_ responses. The dendrogram analysis was suggestive of major differences between embryo and endosperm under ABA and H_2_O_2_ treatment during seed germination. The differential expression analysis between CK-treated and ABA/H_2_O_2_-treated tissues identified a number of differentially expressed genes in the two tissues under different treatments. The differentially expressed genes in embryo under ABA and H_2_O_2_ treatments were closely related to DNA synthesis, CHO metabolism, hormone metabolism, and protein degradation, while those in endosperm under ABA and H_2_O_2_ treatments were related mainly to the metabolism of storage reserves, transport, biotic and abiotic stresses, hormone metabolism, cell wall metabolism, signaling, and development. Scatter plot analysis showed that regulation patterns in the ABA-treated group were similar between the two tissues, while the H_2_O_2_-treated group showed greater expression differences. MapMan analysis provided a global view of the changes in several important metabolic processes (e.g., energy reserve mobilization, cell wall metabolism, and photosynthesis) and functional groups (e.g., cellular processes, hormones and signaling and transport) in embryo and endosperm following exposure to ABA and H_2_O_2_ treatment during germination. qRT-PCR analysis was used to validate the expression patterns of nine genes. Our results provide novel insights into the mechanisms of transcriptional regulation in embryo and endosperm in response to ABA and H_2_O_2_ treatments during seed germination.

## Methods

### Plant material and treatments

Arrays were performed on isolated embryo and endosperm tissues from Zhengmai 9023, an elite Chinese bread wheat cultivar (*Triticum aestivum* L.) with high yield performance and superior quality [68]. Seeds were germinated on wet filter paper in Petri dishes with three biological replicates, and incubated at 25 °C in a growth chamber in the dark. Tissues were harvested from seeds under 100 μmol H_2_O_2_ and 100 mg/L ABA treatment, respectively until radicles just break through the sporniodem. Embryo and endosperm samples were collected by manual dissection as described, and stored in RNAlater solution prior to RNA extraction (Qiagen). Three biological replicates for two tissues under ABA and H_2_O_2_ treatments were used for microarray hybridization.

### RNA isolation and microarray hybridization

Total RNA was extracted from materials using the Trizol® Plus RNA Purification Kit (Invitrogen, Carlsbad, CA) with an on-column DNase treatment. Purified total RNA samples were quantified with Agilent 2100Bioanalyzer (Agilent Technologies, Palo Alto, CA), and satisfactory purity was indicated by A260:280 ratios about 2.0 in 10 mM Tris–HCl (pH 7.5). Integrity of total RNA samples was assessed by denaturing formaldehyde gel electrophoresis, where the presence of sharp 28S and 18S ribosomal RNA bands at an intensity ratio of ~2:1 (28S:18S) indicated good integrity. After that, high quality RNAs can be used to the subsequent high-throughput experiments. Total RNAs were incubated with OligodT/T7 primers and reverse-transcribed into double-stranded cDNA. The amplified RNAs were purified and labeled by biotin with Affymetrix’s IVT labelingkit. The biotinylated cDNAs were fragmented and hybridized to the Affymetrix GeneChip^*®*^ Wheat Genome Array (Affymetrix, Inc., Santa Clara, CA) for 16 h. The wheat genome array includes 61,127 probe sets representing 55,052 transcripts for all 21 wheat chromosomes in the genome. 59,356 probes sets represent modern hexaploid (A, B and D genomes) bread wheat (*T. aestivum*) and are derived from the public content of the *T. aestivum* UniGene Build #38. 1215 probe sets are derived from ESTs of a diploid near relative of the A genome (*T. monococcum*), a further 539 represent ESTs of the tetraploid (A and B genomes) macaroni wheat species *T. turgidum*, and five are from ESTs of a diploid near relative of the D genome known as *Aegilops tauschii*. After washing and staining, the results were scanned and recorded.

### Data treatments and significant differential gene analysis

The microarray imaging data were analyzed with Microarray suite version 5.0 (Affymetrix Inc.), followed by Spotfire (Spotfire, Somerville, MA). Three biological replicates per treatment were hybridized independently to the Affymetrix ATH1 array, washed, stained, and scanned following the procedures described in the Affymetrix technical manual. The expression levels of genes were measured by detection calls and signal intensities using the Micro Array Suite 5.0 software with a target signal of 100. Sixty four Affymetrix controls and 5623 wheat genes that are detected as absent in all 18 chips were removed from the 22,810 probe sets. All microarray data from three biological replicates obtained in this study have been deposited in the NCBI GEO database, which are accessible through GEO Series accession number GSE64030 (https://www.ncbi.nlm.nih.gov/geo/query/acc.cgi?acc=GSE64030). All pairwise differentially expressed genes were identified using SAM software using the data of all the remaining 17,123 wheat probe sets. A false discovery rate parameter of 1 % was used for the SAM analysis. Following SAM analyses, genes that were called absent more than twice among three replicas in both control and treatment arrays were regarded as not expressed in both conditions and then removed from the above list. Z-score transformation was performed as described [69]. This transformation normalizes the data according to the distance of each log10 value from the mean log10 value, expressed in terms of number of standard deviations.

For the MapMan analysis, input files were created by calculating the natural log ratio of the mean detection of the three control samples to the mean detection in the treatment samples. Genes called absent in two out of the three replicates were regarded as not expressed under that particular experimental condition. Final analyses were performed with MapMan version 1.6.1, including automatic application of the Wilcoxon rank sum test [70]. Comparison with public domain Affymetrix ATH1 data sets was achieved by downloading entire data sets from NascArrays and from Nakabayashi et al. [36]. Probe sets were identified that exhibited two fold or greater changes in expression in response to H_2_O_2_ and ABA treatments.

We did the hierarchical clustering to analyze the gene expression profile based on methods described by Eisen et al. [71]. A software named cluster3 was used to do the clustering analysis. The parameters are following: % present is set to > =80, sd is 2, all ratio values are log transformed (base 2 for simplicity), we also selected the median and normalize, then the Euclidean distance similarity metric was used to define the similarity and the hierarchical clusters were assembled using the complete linkage clustering method, the k-means was default.

### Quantitative real-time polymerase chain reaction (qRT-PCR)

Representative differentially expressed genes were verified by qRT-PCR. After RNA isolation, first-strand cDNA was synthesized in a 20-μl volume containing 0.5 μl AMV reverse transcriptase (Promega), 0.5 μl RNase inhibitor (Promega), 1 μl oligodT primer, 2 μl dNTP mixture, 4 μl MgCl2 (25 mM), 2 μl 10 × reverse transcriptase buffer and 4 μl RNA sample. The reaction mixture was incubated at 42 °C for 60 min.

Double standard curve method was used to detect the gene expression levels. ADP-ribosylation factor was used as the internal control, which was identified as one of the most stably expressed genes [72]. Gene-specific primers were designed using Primer 5.0, and their specificities were checked by the melting curves of the RT-PCR products. Each qRT-PCR reaction was performed in 20-μl volumes containing 10 μl 2 × SYBR Premix Ex Taq (TaKaRa), 2 μl 50-fold diluted cDNA, 0.4 μl of each gene-specific primer, and 7.2 μl ddH_2_O. PCR conditions were as follows: 95 °C for 3 min, 45 cycles of 15 s at 95 °C, 57 °C for 15 s and 72 °C for 20 s. Three replicates were used for each sample. Reactions were conducted in a CFX96 Real-Time PCR Detection System (Bio-Rad). All data were analyzed with CFX Manager Software (Bio-Rad).

### Availability of supporting data

The datasets supporting the results of this article are included within the article and its Additional files. All microarray data from three biological replicates obtained in this study have been deposited in the NCBI GEO database, which are accessible through GEO Series accession number GSE64030 (https://www.ncbi.nlm.nih.gov/geo/query/acc.cgi?acc=GSE64030).

### Ethics approval and consent to participate

Not applicable.

### Consent for publication

Not applicable.

## Additional files

Additional file 1: Table S1. Complete list of normalized expression values obtained from three biological replicates based on independently treated plant materials. Each horizontal row represents an individual probe set; information of every probe set are represented in vertical columns, including the corresponding functional group of every probe set and their normalized expression values (ABA/H_2_O_2_ vs CK in different tissues). (xlsx 5.77 MB) Additional file 2: Table S2. The list of significant differentially expressed genes under ABA and H_2_O_2_ treatment respectively in the different tissues (embryo and endosperm). ABA vs CK_embryo: ABA_embryo compared to CK_embryo; H_2_O_2_ vs CK_embryo: H_2_O_2__embryo compared to CK_ embryo; ABA vs CK_endosperm: ABA_endosperm vs CK_endosperm; H_2_O_2_ vs CK_endosperm: H_2_O_2_ _endosperm compared to CK_endosperm. (xls 728 KB) Additional file 3: Figure S1. Hierarchical clustering of genes. Heat map of hierarchical clustering for significant differentially expressed genes: horizontal rows represent individual genes and vertical rows represent different treatment. Red and green indicate transcript level above and below the median for that gene across all samples, respectively. Distinct clusters of significant differentially expressed genes can be seen for ABA, H_2_0_2_ treatment compared to control. (tif 3.49 MB) Additional file 4: Table S3. Primer sequences used for qRT-PCR analysis. (xlsx 10.8 KB) Additional file 5: Figure S2. The standard curves and melt peaks of the targeted genes. (pdf 1.20 MB)

## Abbreviations

ABA: Abscisic acid
BR: Brassinosteroids
CK: Contrast check
DEGs: Differentially expressed genes
FDR: False discovery rate
GA: Gibberelin
H_2_O_2_: Hydrogen peroxide
IAA: Auxin
JA: Jasmonate
MAPK: Mitogen-activated protein kinases
PK: Pyruvate kinase
PPFK: Phosphofructokinase
qRT-PCR: Quantitative real-time polymerase chain reaction
ROS: Reactive oxygen species
SAM: Significance analysis of microarrays
SUSY: Sucrose synthase
UGPase: UDP-glucose pyrophosphorylase
