# Supplementary figures and images for: Comparative transcriptome analysis of wheat embryo and endosperm responses to ABA and H2O2 stresses during seed germination
Source: BMC Genomics. 2016 Feb 4;17:97. doi: 10.1186/s12864-016-2416-9 (PMC4743158; doi:10.1186/s12864-016-2416-9)

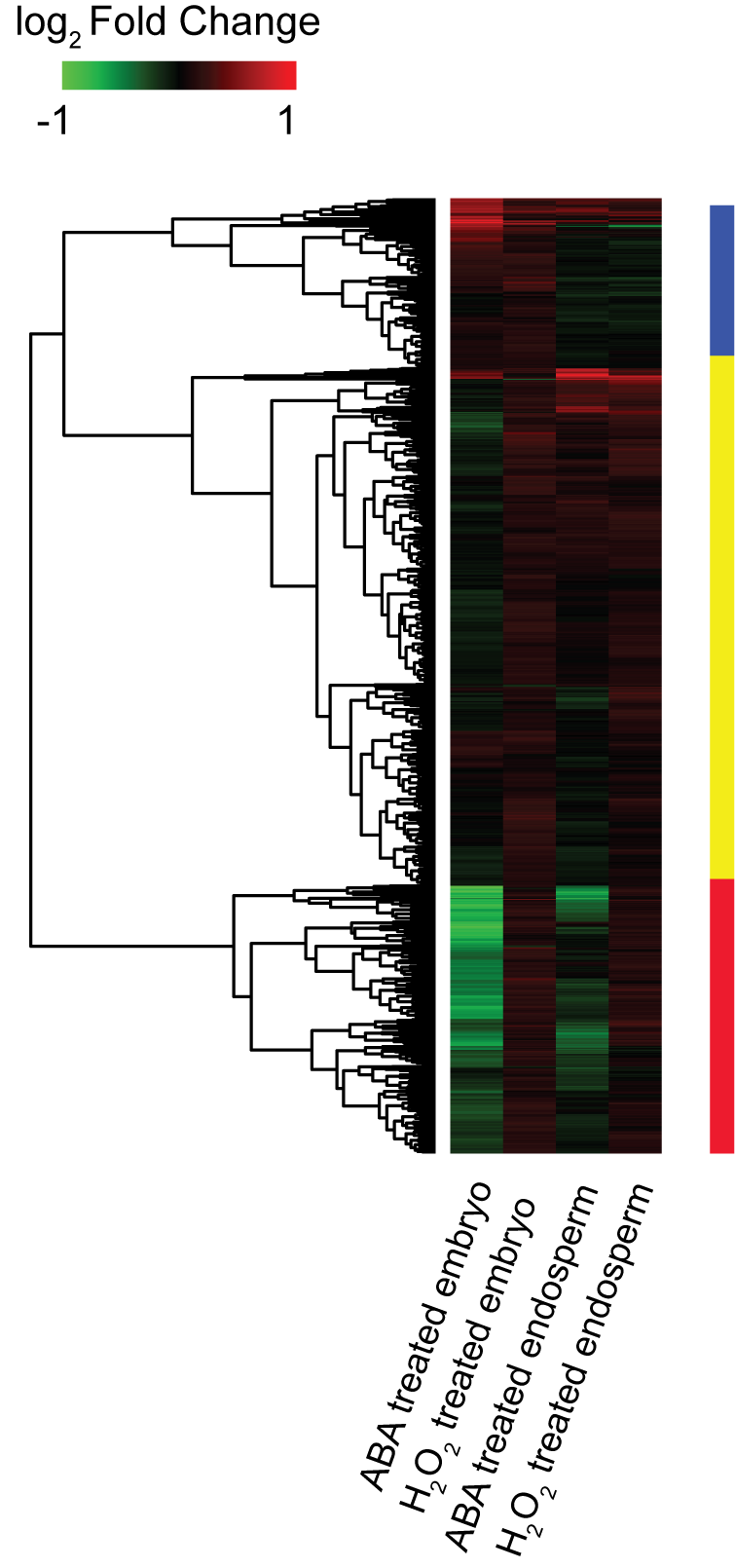

Supplement: Additional file 3: Figure S1. — Hierarchical clustering of genes. Heat map of hierarchical clustering for significant differentially expressed genes: horizontal rows represent individual genes and vertical rows represent different treatment. Red and green indicate transcript level above and below the median for that gene across all samples, respectively. Distinct clusters of significant differentially expressed genes can be seen for ABA, H202 treatment compared to control. (tif 3.49 MB) [file 12864_2016_2416_MOESM3_ESM.tif]
