# Supplementary material for: Comparative transcriptome analysis of wheat embryo and endosperm responses to ABA and H2O2 stresses during seed germination
Source: BMC Genomics. 2016 Feb 4;17:97. doi: 10.1186/s12864-016-2416-9 (PMC4743158; doi:10.1186/s12864-016-2416-9)
Supplement: Additional file 5: Figure S2. — The standard curves and melt peaks of the targeted genes. (pdf 1.20 MB) [file 12864_2016_2416_MOESM5_ESM.pdf]

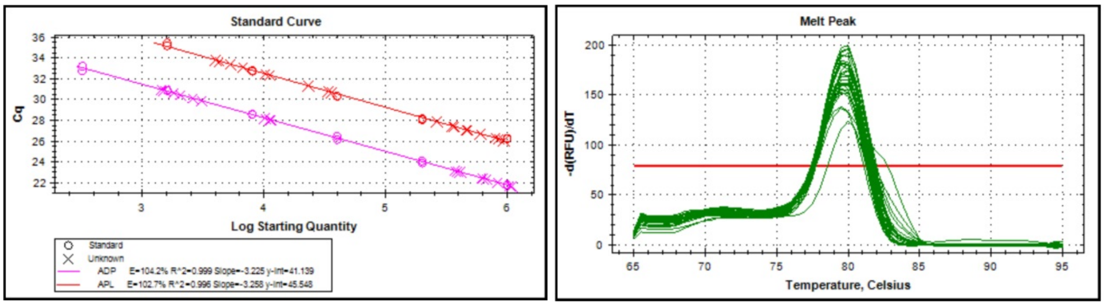

Glucose-1-phosphate adenylyltransferase

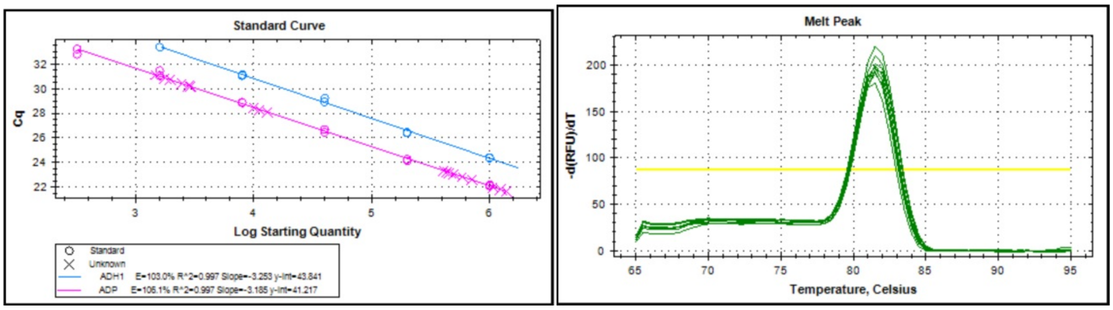

Alcohol dehydrogenase

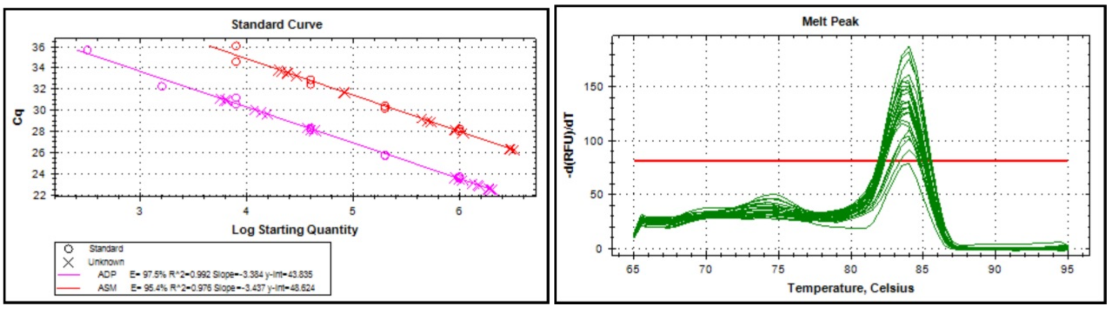

DNA binding / transcription factor

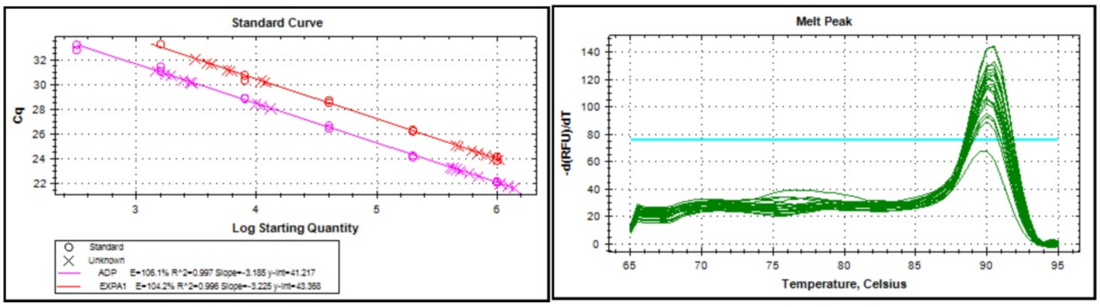

Expansin A1

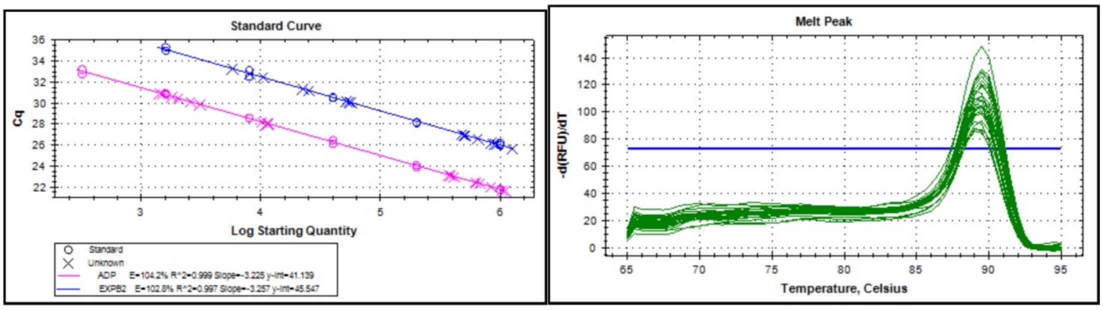

Expansin B2

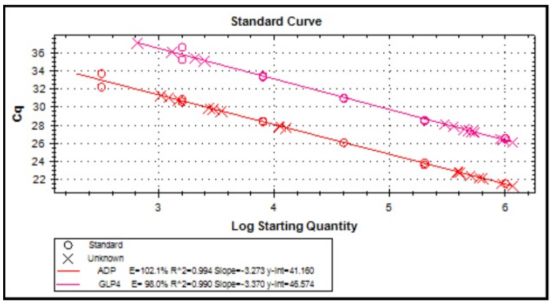

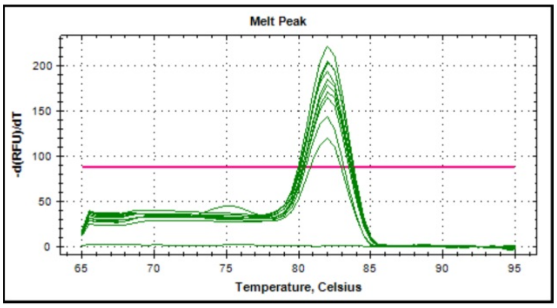

Germin-like protein 4

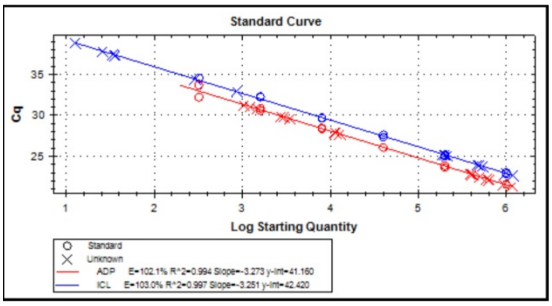

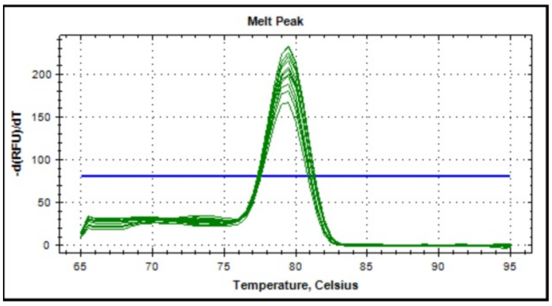

Nucleotide-sensitive chloride conductance regulator family protein

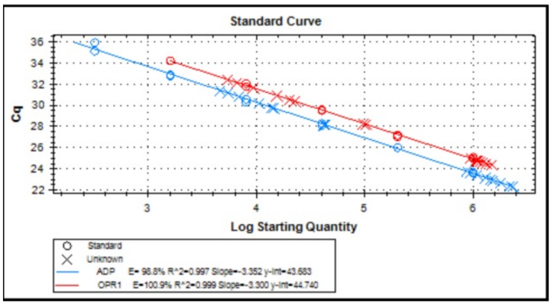

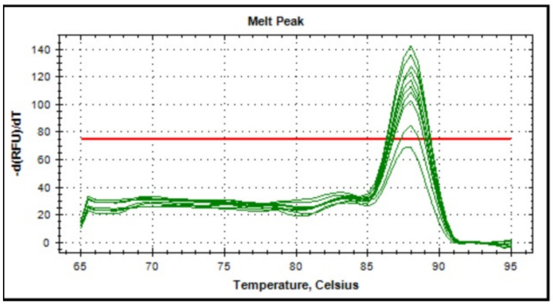

12-oxophytodienoate reductase 1

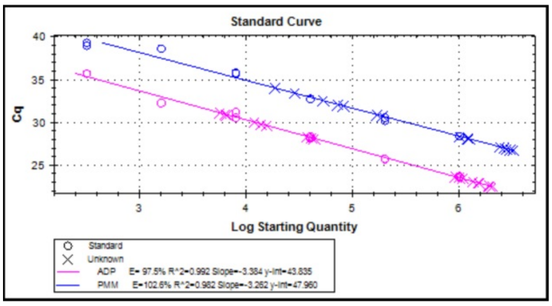

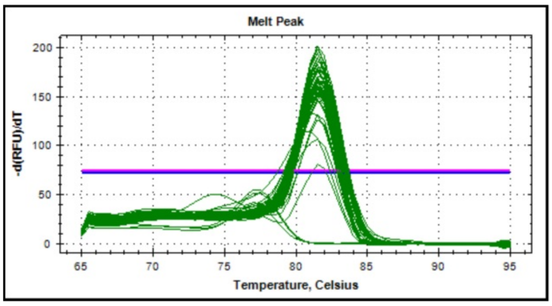

Phosphomannomutase
